# Phosphonium ionic liquids as extractants for recovery of ruthenium(III) from acidic aqueous solutions

**DOI:** 10.1007/s11696-016-0027-1

**Published:** 2016-12-21

**Authors:** Martyna Rzelewska, Monika Baczyńska, Maciej Wiśniewski, Magdalena Regel-Rosocka

**Affiliations:** Faculty of Chemical Technology, Institute of Chemical Technology and Engineering, Poznan University of Technology, ul. Berdychowo 4, 60-965 Poznan, Poland

**Keywords:** Liquid–liquid extraction, Ruthenium(III), Rhodium(III), Phosphonium ionic liquid, Cyphos IL 101, Cyphos IL 104, Cyphos IL 167

## Abstract

The aim of this work is to investigate extraction of ruthenium(III) from acidic aqueous solutions with phosphonium ionic liquids such as trihexyl(tetradecyl)phosphonium chloride (Cyphos IL 101), trihexyl(tetradecyl)phosphonium *bis*(2,4,4-trimethylpentyl)phosphinate (Cyphos IL 104) and tributyl(tetradecyl)phosphonium chloride (Cyphos IL 167) as extractants. The influence of HCl content in the feed solutions on extraction of Ru(III) was investigated. The research was performed for model solutions containing Ru(III) and a mixture of waste solutions containing Ru(III) and Rh(III). In addition, investigation of the type of extractant and its concentration in the organic phase on extraction of Ru(III) was carried out. Co-extraction of protons to the organic phase was determined. To the best of our knowledge, the extraction of Ru(III) with Cyphos IL 167 (tributyl(tetradecyl)phosphonium chloride) as an extractant has not yet been described in the scientific literature.

## Introduction

The Platinum Group Metals (PGM) such as rhodium, ruthenium, palladium and platinum are extremely rare in nature comparing to other metals. Natural ores contain small amounts of PGM; therefore, their cost of production is very high. All over the world, PGM are mined mainly in South Africa (The Bushveld Complex), Russia (The Urals deposits), North America (Canada) and Zimbabwe (The Great Dyke) (Matthey 2016). The technological process of platinum ore processing is energy-consuming and also causes environment-damaging activities. On a global scale, 31% production of Pt, 57% production of Pd and 84% production of Rh are used to produce automotive catalytic converters. Currently, secondary sources such as waste electrical and electronic equipment and catalytic converters become the main source of PGM (Fornalczyk and Saternus 2007).

Due to the large interest in these metals, new methods of PGM recovery are developed. A popular processing of PGM from the secondary sources covers leaching and reactive extraction. At first, waste material, mainly spent petroleum and automotive catalysts containing PGM and non-precious metals such as Cu, Fe, Mg, Ca, Zn and Bi, is leached with concentrated hydrochloric acid (Dragulovic et al. 2011; Pośpiech 2012; Sun and Lee 2013). After leaching, a pregnant leach solution (PLS) with desirable ion metals is contacted with organic phase containing an extractant to recover valuable metals from PLS.

A reactive extraction is a simple and efficient technique for separation of metal ions from diluted effluents. In addition, the reactive extraction is the most popular separation technique of metal ions among hydrometallurgical methods but the use of organic compounds (many times volatile organic compounds) is indicated as its drawback.

For over 15 years, ionic liquids (ILs) have been established as popular and efficient extractants or solvents in separation processes (Abbott et al. 2011; Billard, 2013; Regel-Rosocka and Materna 2014). Ionic liquids, such as phosphonium ILs, can be used as extractants for liquid–liquid extraction of organic compounds (Marták and Schlosser 2004, 2006, 2016), lanthanides (Kumari et al. 2016), heavy metals (Guo et al. 2011; Leyma et al. 2016; Regel-Rosocka et al. 2006, 2012; Vander Hoogerstraete et al. 2013) or PGMs (Cieszynska and Wisniewski 2010, 2012; Papaiconomou et al. 2015; Regel-Rosocka et al. 2015; Rzelewska et al. 2016). They are also applied as carriers of metal ions in polymer inclusion membranes (Baczyńska et al. 2016; Baczyńska and Regel-Rosocka 2013; Kogelnig et al. 2011; Pośpiech 2015; Regel-Rosocka et al. 2012, 2015) or supported liquid membranes (Alguacil et al. 2010; de San et al. 2014) and in sorption as Cyphos-impregnated resins or biopolymer capsules (Guibal et al. 2008; Navarro et al. 2016; Vincent et al. 2008).

The main interest in PGM extraction is focused on the separation of Pd(II) and Pt(IV) from HCl solutions (Cieszyńska and Wiśniewski 2010, 2012; Papaiconomou et al. 2015), while extraction systems for Ru(III) and Rh(III) separation are scarcely described. Selective extraction of Ru(III) in the presence of Ir(IV) and Rh(III) (Góralska et al. 2007) with Alamine 336 or in the presence of Os(IV) and Pt(IV) with thiourea derivative (Kuchekar et al. 2015) was studied. In addition, an analytical application of 4-pyridone derivatives for Ru(III) extraction from large amounts of Pd(II) and Rh(III) was proposed (Druškovic et al. 2005).

Looking for selective extractants to separate Ru(III) from Rh(III) the authors of the paper propose to use phosphonium ILs. The problem of separation of these metal ions is important because of scarce natural ores and increasing demand for them. The aim of this work is to investigate the effect of HCl content in the feed on extraction of ruthenium(III) from acidic aqueous solutions with phosphonium ionic liquids such as trihexyl(tetradecyl)phosphonium chloride (Cyphos IL 101), trihexyl(tetradecyl)phosphonium *bis*(2,4,4-trimethylpentyl)phosphinate (Cyphos IL 104) and tributyl(tetradecyl)phosphonium chloride (Cyphos IL 167) as extractants. It is important to mention that also selective extraction of Ru(III) in the presence of Rh(III) was investigated.

## Experimental

### Chemicals

Phosphonium ionic liquids, trihexyl(tetradecyl)phosphonium chloride (Cyphos IL 101), trihexyl(tetradecyl)phosphonium *bis*(2,4,4-trimethylpentyl)phosphinate (Cyphos IL 104) and tributyl(tetradecyl)phosphonium chloride (Cyphos IL 167) supplied by Cytec Industries Inc., were dissolved in toluene (5 × 10^−3^, 2 × 10^−2^ and 5 × 10^−2^ M). The structures of the ILs applied are presented in Table 1.

**Table 1:**
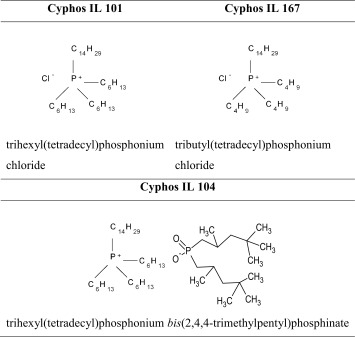
Structures of the ILs

A twofold excess of phosphonium IL in the organic phase to Ru(III) content in the feed was taken into consideration in all the presented extraction studies. The model aqueous solutions contained 2.5 × 10^−3^ M Ru(III) in 0.1, 1, 3 and 5 M HCl. Another feed solution contained mixture of Ru(III) and Rh(III) containing 1.7 × 10^−3^ M Ru(III) and 0.5 × 10^−3^ M Rh(III) in 2.5 M HCl with 2.9 M NaCl. In addition, an effect of IL concentration in the organic phase on extraction of metal ions was studied.

### Methods and analytical equipment

Extraction was carried out in glass separatory funnels for 20 min at 22 ± 2 °C. Feed solutions containing 2.5 × 10^−3^ M Ru(III) in various concentrations of HCl or mixture of: 1.7 × 10^−3^ M Ru(III) and 0.5 × 10^−3^ M Rh(III) in 2.5 M HCl with 2.9 M NaCl were mechanically shaken with IL-containing phase (volume ratio w/o = 1) and then allowed to stand for phase separation. Stripping of Ru(III) ions from the loaded organic phases was carried out with 0.1 M thiourea in 0.5 M HCl. The loaded organic phase and the stripping phase were shaken at volume ratio w/o = 1 for 20 min at 22 ± 2 °C in glass separatory funnels and then allowed to stand for phase separation. Atomic absorption spectrometer Hitachi Z-8200 was used for determination of Rh(III) and Ru(III) in the aqueous solutions at 369.2 and 349.9 nm, respectively. HCl concentration in the aqueous phases was determined by potentiometric titration with 0.1 M NaOH (702 SM, Metrohm).

## Results and discussion

### Influence of HCl concentration in Ru(III) extraction

Effect of concentration of HCl in the feed solution on Ru(III) extraction was studied and the results are shown in Table 2. Both phases (the feed solution and the IL-containing organic phase) were shaken together for 20 min. Distribution ratio D_Ru(III)_ was defined as the ratio of Ru(III) concentrations in the organic C*_(org)_ and the aqueous C*_(aq)_ phases after extraction (IUPAC 2014).

 The highest distribution ratio of Ru(III) is obtained for IL 167 as an extractant and reaches even more than 2.7 (for 1 M HCl in the feed). D_Ru(III)_ values amount to 0.26–0.35, and are the smallest for all ILs when the concentration of HCl in the feed solution is 5 M. The values presented in Table 2 indicate that Cyphos IL 167 extracts Ru(III) from low HCl-concentrated feeds much better than Cyphos IL 101 and IL 104. However, relatively short carbon chains at tributyl(tetradecyl)phosphonium cation make Cyphos IL 167 more hydrophilic than Cyphos IL 101. Hence, formation of emulsions and very slow and difficult phase separation are noted for this IL. The emulsions after extraction were left for 24 h to complete separation and to analyze Ru(III) and Rh(III) extraction. This is the reason for elimination of IL 167 from our studies in the future.

**Table 2:** Distribution ratio of Ru(III) between Cyphos IL 101, IL 104 or IL 167-containing organic phases and aqueous solutions with various concentrations of HCl

HCl concentration/M	**D**_**Ru(III)**_		
	Cyphos IL 101	Cyphos IL 104	Cyphos IL 167
0.1	1.48 ± 0.09	1.44 ± 0.09	2.28 ± 0.18
1	2.28 ± 0.27	2.10 ± 0.27	2.72 ± 0.18
3	2.62 ± 0.45	1.65 ± 0.18	2.00 ± 0.36
5	0.35 ± 0.09	0.26 ± 0.00	0.27 ± 0.18

(Organic phase: 5 × 10^−3^ M IL in toluene; feed phase: 2.5 × 10^−3^ M Ru(III) in 0.1–5 M HCl)

Increase in HCl concentration in the feed causes positive changes in D_Ru(III)_ up to certain HCl content, after reaching maximum value of *D*_Ru(III)_ (2.62 at 3 M HCl with Cyphos IL 101 and 2.10; 2.72 at 1 M HCl for Cyphos IL 104 and IL 167, respectively) the distribution of Ru(III) into the organic and the aqueous phases decreases. This negative effect of HCl presence in the feed on efficiency of Ru(III) extraction is likely to be caused by co-extraction of the acid into the organic phase, which is particularly visible for the feed containing 5 M HCl.

Therefore, concentration of hydrochloric acid before and after Ru(III) extraction was determined in the aqueous phases, and the results of HCl transport to the organic phase are shown in Table 3. Additionally, transport of the acid from the feeds without Ru(III) was studied and is shown also in Table 3.

**Table 3:** Hydrochloric acid extraction to the organic phases of 5 × 10^−3^ M Cyphos IL 101, Cyphos IL 104 or Cyphos IL 167

HCl/M	After extraction with IL 101		After extraction with IL 104		After extraction with IL 167	
	HCl_aq_/M	HCl_org_/M	HCl_aq_/M	HCl_org_/M	HCl_aq_/M	HCl_org_/M
Feed: 2.5 × 10^−3^ M Ru(III) in 0.1–5 M HCl						
0.1	0.094	0.004	0.096	0.002	0.097	0.012
1	0.983	0.006	0.970	0.019	0.897	0.019
3	2.767	0.042	2.751	0.050	2.640	0.067
5	4.554	0.047	4.545	0.056	4.394	0.126
Feed: aqueous solutions of 0.1–5 M HCl						
0.1	0.096	0.007	0.097	0.006	0.102	0.001
1	0.942	0.005	0.945	0.002	0.928	0.016
3	2.864	0.027	2.872	0.019	2.815	0.076
5	4.919	0.291	5.008	0.202	4.877	0.334

The amount of HCl transported grows with its increasing content in the feeds both with Ru(III) and without metal ions. Significant co-extraction of HCl and Ru(III), observed at high concentration of the acid (5 M), explains decrease in the value of D_Ru(III)_ with increasing HCl content in the feed. However, the content of HCl after the extraction from 5 M feed exceeds significantly the IL concentration which cannot be assigned only to reaction of HCl extraction with IL as it was proposed by Góralska et al. (2007) for quaternary ammonium salts:

1 $${\rm R}_{3}^{{}} {\rm NHCl}_{({\rm org})} + {\rm H}_{({\rm aq})}^{ + } + {\rm Cl}_{({\rm aq})}^{ - } \Leftrightarrow {\rm R}_{3} {\rm NH}_{2} {\rm Cl}_{2({\rm org})}$$

As Martak and Schlosser (2006, 2016) emphasized, phosphonium ILs are prone to form reverse micelles in extraction systems. Thus, excessive HCl can be transported to the organic phase incorporated into reverse micelles. It is clearly visible for IL 167, which extracts high amount of HCl even in the presence of Ru(III) and forms emulsions as a result of higher hydrophilicity of the phosphonium cation compared to Cyphos IL 101.

Chemistry of Ru(III) species in chloride solutions is complex. However, the literature data (Afzaletdinova et al. 2007; Orysyk et al. 2010) show that at Cl^−^ concentration below 6 M, apart from [Ru(H_2_O)Cl_5_]^2−^, the solution contains also [Ru(H_2_O)_2_Cl_4_]^−^. Moreover, some amounts of [Ru(H_2_O)_3_Cl_3_], *cis*- and *trans*-[Ru(H_2_O)_4_Cl_2_]^+^, and [Ru(H_2_O)_5_Cl]^2+^ ions can be present. For anionic chlorocomplexes of Ru(III), the mechanism of extraction with Cyphos ILs is similar to that one proposed by Cieszyńska and Wiśniewski (2012, 2012) for Pd(II) extraction. Phosphonium chlorides, similarly to quaternary ammonium salts (Panigrahi et al. 2014), can extract trivalent metal chlorocomplexes according to anion-exchange reactions:

2 $${\rm RuCl}_{5({\rm aq})}^{2 - } + 2{\rm R}_{3} {\rm R}'{\rm PCl}_{({\rm org})} \Leftrightarrow ({\rm R}_{3} {\rm R}'{\rm P})_{2} {\rm RuCl}_{5({\rm org})} + 2{\rm Cl}_{({\rm aq})}^{ - }$$

3 $${\rm RuCl}_{4({\rm aq})}^{ - } + {\rm R}_{3}^{{}} {\rm R}'{\rm PCl}_{({\rm org})} \Leftrightarrow ({\rm R}_{3}^{{}} {\rm R}'{\rm P}){\rm RuCl}_{4({\rm org})} + {\rm Cl}_{({\rm aq})}^{ - }$$

Moreover, as Cyphos IL 104 consists of *bis*(2,4,4-trimethylpentyl)phosphinate (originating from the well-known acidic extractant Cyanex 272) and is proven to form the organophosphoric acid in the organic phase during extraction of Pd(II) (Cieszyńska and Wiśniewski 2010, 2012) the extraction of Ru(III) is likely to run as follows:

4 $${\rm H}_{({\rm aq})}^{ + } + {\rm RuCl}_{4({\rm aq})}^{ - } + {\rm R}_{3}^{{}} {\rm R}'{\rm PCl}_{({\rm org})} \Leftrightarrow ({\rm R}_{3}^{{}} {\rm R}'{\rm P}){\rm RuCl}_{4({\rm org})} + {\rm HA}_{({\rm org})}^{{}}$$

where A stands for *bis*(2,4,4-trimethylpentyl)phosphinate anion, subscripts (org) and (aq) denote the organic and the aqueous phases, respectively.

### Stripping of Ru(III) from the loaded organic phases

The organic phases loaded with Ru(III), as a result of extraction from 0.1 to 5 M HCl, were stripped with solution of 0.1 M thiourea in 0.5 M HCl. The results for stripping of Ru(III) are presented in Table 4.

**Table 4:** Percentage stripping of Ru(III) from the loaded organic phases containing 5 × 10^−3^ M Cyphos IL 101, IL 104 or IL 167

HCl in feed/M	Cyphos IL 101			Cyphos IL 104			Cyphos IL 167		
	*C*_org_/(mg dm^−3^)	*C*_aq_/(mg dm^−3^)	*R*/%	*C*_org_/(mg dm^−3^)	*C*_aq_/(mg dm^−3^)	*R*/%	*C*_org_/(mg dm^−3^)	*C*_aq_/(mg dm^−3^)	*R*/%
0.1	107.0	100.0	93.5	105.8	91.8	86.8	129.9	119.0	91.6
1	119.3	114.2	95.7	116.3	75.7	65.1	135.9	116.5	85.7
3	128.7	88.4	68.7	111.3	84.9	76.3	130.0	85.9	66.1
5	48.5	30.1	62.1	39.4	28.3	71.8	42.1	38.0	90.4

*C*_org_—concentration of Ru(III) in the organic phase after extraction, C_aq_—concentration of Ru(III) in the stripping phase (strippant) and *R*—percentage stripping

About 100–135 mg dm^−3^ of Ru(III) is extracted to the organic phase after 20 min of extraction from feeds containing 0.1–3 M HCl. When the feed contains 5 M HCl only 40–48 mg dm^−3^ of Ru(III) is transported to the organic phase after extraction. Ru(III) stripping with 0.1 M thiourea in 0.5 M HCl is very efficient from all ILs loaded with Ru(III) at low content of HCl. Stripping efficiency of Ru(III) from the organic phase equals about 90%.

### Influence of shaking time on extraction of Ru(III) and Rh(III) from their mixture

An important issue for separation processes is the possibility to recover selectively some required species from their mixtures. In this paper, separation of Ru(III) from Rh(III) using one of three ionic liquids as extractant was studied. The feed contained mixture of Ru(III) and Rh(III): 1.7 × 10^−3^ M Ru(III) and 0.5 × 10^−3^ M Rh(III) in 2.5 M HCl with 2.9 M NaCl.

**Fig. 1:**
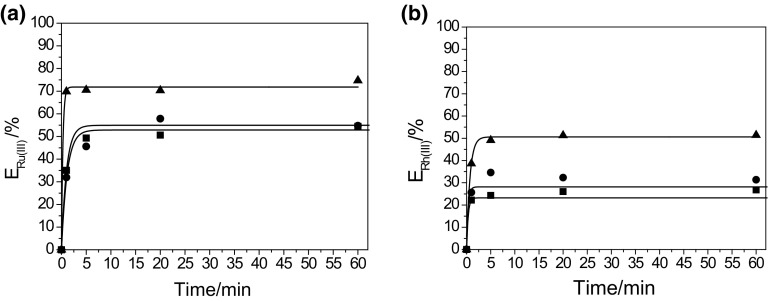
Effect of shaking time on the extraction of **a** Ru(III) and **b** Rh(III) from the mixture of these metal ions (Organic phase: 5 × 10^−3^ M (*filled square*) IL 101; (*filled circle*) IL 104; (*filled triangle*) IL 167 in toluene)

At first, for the mixtures of Ru(III) and Rh(III), an effect of contact time of both phases (the aqueous and the organic) on the extraction of metal ions from their mixture was investigated and the results are shown in Fig. 1.

As it can be seen from Fig. 1, the extraction equilibrium for both Ru(III) and Rh(III) is reached after 20 min. The most effective extractant for Ru(III) from mixture of the metal ions is Cyphos IL 167; extraction efficiency of Ru(III) is equal to about 70%. Extraction efficiency of Rh(III) is small regardless of the type of the extractant and amounts to about 50%. The extraction efficiency of Ru(III) and Rh(III) decreases in the following order: Cyphos IL 167 > Cyphos IL 104 > Cyphos IL 101.

### Influence of Cyphos IL concentration on extraction of Ru(III) and Rh(III) from their mixture

For model studies, two-component solutions were prepared by mixing one-component feeds and raffinates from our previous studies to investigate the possibility of selective separation of Ru(III) from Rh(III). Generally, concentration of HCl in the PGM leaching solutions reported by different authors changes in the range from 1 even up to 12 M HCl. These are solutions after leaching of PGM concentrates (Bernardis et al. 2005; Jha et al. 2014) or secondary sources such as automotive and industrial catalytic converters (Jimenez de Aberasturi et al. 2011; Suoranta et al. 2015). The model solutions used by us were the mixture of various aqueous solutions of HCl with concentration close to 3 M.

**Fig. 2:**
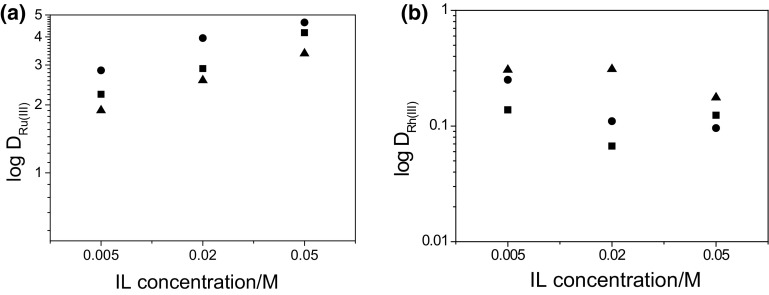
Effect of ILs concentration in the organic phase on extraction of **a** Ru(III) and **b** Rh(III) from the mixture of these metal ions (Organic phase: (*filled square*) IL 101; (*filled circle*) IL 104; (*filled triangle*) IL 167 in toluene)

An effect of ILs’ concentration in the organic phase on extraction of Ru(III) and Rh(III) from a mixture of these metal ions is presented in Fig. 2.

On the basis of *D* values, the extraction selectivity of Ru(III) in the presence of Rh(III) with Cyphos IL 101, Cyphos IL 104 or Cyphos IL 167 was calculated as the ratio of *D*_Ru(III)_ and *D*_Rh(III)_. The results are shown in Table 5.

**Table 5:** Extraction selectivity of the Ru(III) in the presence of Rh(III) with Cyphos IL 101, Cyphos IL 104 or Cyphos IL 167

IL/M	S_Ru(III)/Rh(III)_		
	Cyphos IL 101	Cyphos IL 104	Cyphos IL 167
5 × 10^−3^	16.4	43.0	33.6
2 × 10^−2^	11.3	35.9	48.1
5 × 10^−2^	6.2	8.3	19.2

It is obvious that all ILs used are selective extractants for Ru(III) recovery from its mixture with Rh(III). Moreover, Cyphos IL 104 is a more efficient extractant of Ru(III) from mixture than Cyphos IL 101 (Fig. 2a; Table 5). The values of Ru(III) distribution ratio (*D*_Ru(III)_) are high, while Rh(III) extraction with all IL-organic phases is poor. In addition, an increase in IL content in the organic phase causes a rise in *D*_Ru(III)_ from about 2.2 to 4.2 (IL 101), 2.8 to 4.6 (IL 104) and 1.9 to 3.4 (IL 167) (Fig. 2a), while *D*_Rh(III)_ does not exceed 0.35 (Fig. 2b) and is not affected by the concentration of IL in the organic phase. Hence, the best selectivity of Ru(III) extraction is obtained with 5 × 10^−3^ and 2 × 10^−2^ M Cyphos IL 104 and IL 167.

## Conclusions

The results presented in the paper indicate that Cyphos IL 167 is the best extractant for extraction of Ru(III) from single-metal feeds. Distribution ratio of Ru(III) is equal to almost 3 for 1 M HCl in the feed; however, the problems with emulsion formation eliminate this IL from application as extractant.

With increasing concentration of HCl in the feed, extraction of HCl into the organic phase also increases. Co-extraction of HCl to the organic phase may be the reason for the decrease in *D*_Ru(III)_ values. *D*_Ru(III)_ are the smallest for all ILs when the concentration of HCl in the feed is 5 M. An excessive transport of HCl in the relation to IL content in the organic phase can be assigned to formation of reverse micelles in the organic phase containing phosphonium ILs.

Stripping efficiency of Ru(III) from the organic phases is equal to about 90%, when the organic phases were loaded with Ru(III) from the feed solutions containing small amounts of HCl.

The presence of NaCl in the feed phase (mixture of Ru(III) and Rh(III)) facilitates the phase separation as a result of the salting out effect. Additionally, it also ensures content of chloride ions high enough to form anionic chlorocomplexes of Ru(III).

All ILs studied are very selective for Ru(III) extraction in the presence of Rh(III). Also it is noteworthy that Cyphos IL 104 is the best extractant for Ru(III) from mixture of Ru(III)–Rh(III). On the contrary, Rh(III) extraction from mixture of Ru(III)–Rh(III) is very low, distribution ratio D_Rh(III)_ does not exceed 0.35. Thus, the best selectivity of Ru(III) extraction is obtained with 5 × 10^−3^ and 2 × 10^−2^ M Cyphos IL 104 and IL 167. Rh(III) extraction does not depend on the concentration of the IL used.
